# *Trichoderma* as a biological control agent against *Colletotrichum*-associated plant diseases: current insights and future perspectives

**DOI:** 10.1080/15592324.2026.2691464

**Published:** 2026-06-19

**Authors:** Rendra Adhitya, Mia Miranti, Thomas Argyarich Jefferson, Malvin Albert, Ravindra Chandra Joshi, Muhamad Shakirin Mispan, Dedat Prismantoro, Nur Syafikah Abdullah, Mehrdad Alizadeh, Wawan Hermawan, Febri Doni

**Affiliations:** a Department of Biology, Faculty of Mathematics and Natural Sciences, Universitas Padjadjaran, Jatinangor, West Java, Indonesia; b Philippine Rice Research Institute, Maligaya, Science City of Munoz, Munoz, Nueva Ecija, Philippines; c Institute of Biological Sciences, Faculty of Science, Universiti Malaya, Kuala Lumpur, Malaysia; d Doctorate Program in Biotechnology, Graduate School, Universitas Padjadjaran, Bandung, West Java, Indonesia; e Centre for Research in Biotechnology for Agriculture (CEBAR), Universiti Malaya, Kuala Lumpur, Malaysia; f Department of Soil Science, University of Tehran, Tehran, Iran

**Keywords:** Biological control, induced systemic resistance, mycoparasitism, secondary metabolites, sustainable agriculture

## Abstract

*Colletotrichum* species are a taxonomically complex group of hemibiotrophic fungi that cause anthracnose diseases, posing a major constraint to global crop productivity. Their broad host range, high genetic diversity, and ability to shift between biotrophic and necrotrophic phases complicate effective disease management. Although synthetic fungicides are widely used, their prolonged application has raised concerns regarding pathogen resistance, environmental contamination, and food safety, driving the search for sustainable approaches. Unlike previous reviews that mainly discuss *Colletotrichum* biology or *Trichoderma* biocontrol mechanisms separately, this review integrates pathogen taxonomy and infection biology with mechanistic, applied, and future-oriented perspectives on *Trichoderma*-based anthracnose management. Evidence from laboratory, greenhouse, field, and postharvest studies indicates that *Trichoderma* has promising potential for suppressing *Colletotrichum*, particularly under controlled conditions, although its efficacy remains influenced by environmental factors, strain specificity, formulation stability, and application method. Integrating *Trichoderma*-based products into disease management systems offers a promising strategy to reduce chemical inputs and enhance crop resilience. Future research should focus on multi-omics approaches, strain optimization, formulation development, and field validation to ensure consistent performance across diverse agroecosystems.

## 1. Introduction

*Colletotrichum* infects more than 3,200 plant species, including major food, horticultural, and plantation crops, making it one of the most destructive fungal genera worldwide.^1-4^ It causes anthracnose in crops such as chili, strawberry, tomato, mango, coffee, cacao, and rubber, particularly in tropical and subtropical regions where environmental conditions favor disease development.^4-7^ The pathogen follows a hemibiotrophic lifestyle, initially colonizing host tissues biotrophically before shifting to a destructive necrotrophic phase marked by acervuli formation and rapid conidial spread.^8-11^ Disease losses can reach 50–80%, especially under postharvest conditions where latent infections develop during storage and distribution.^11-13^ This broad host range, combined with its capacity to infect diverse plant organs, positions *Colletotrichum* as a major global threat to agricultural productivity.

The genus *Colletotrichum* comprises a highly diverse and economically important group of plant-pathogenic fungi classified into several species complexes, including *C. gloeosporioides*, *C. acutatum*, *C. orbiculare*, and *C. musae*.^3^ ^,^ ^4^ ^,^ ^14^ Because morphological traits often overlap among species, modern taxonomy relies on multilocus phylogenetic analyzes using markers such as ITS, GAPDH, CHS-1, ACT, and TUB2 to delineate species boundaries accurately. Phylogenomic studies have further revealed numerous cryptic species that were previously indistinguishable based on phenotype alone, improving pathogen identification and supporting more precise anthracnose management across crops.^4^ ^,^ ^14^ ^,^ ^15^ Collectively, the taxonomic complexity and hidden diversity of *Colletotrichum* underscore the need for deeper biological understanding to manage the diseases it causes effectively.

Synthetic fungicides remain the primary strategy for managing *Colletotrichum* spp., with combinations such as fluopyram and tebuconazole significantly reducing disease severity when properly applied.^4^ ^,^ ^16^ However, intensive fungicide use can promote resistant pathogen populations, increase environmental contamination, and disrupt beneficial microbial communities in agroecosystems.^1^ ^,^ ^6^ ^,^ ^17^ These concerns have accelerated the search for sustainable alternatives, including antagonistic microorganisms and plant-derived compounds for preharvest and postharvest disease management.^18-20^ Therefore, the limitations of chemical-based control highlight the need to develop environmentally sustainable and biologically driven strategies for long-term management of *Colletotrichum*-associated diseases.

Biological control using beneficial fungi offers a sustainable alternative for reducing chemical dependence while enhancing plant resilience. Antagonistic fungi such as *Trichoderma* spp., *Beauveria bassiana*, and *Aureobasidium pullulans* have been investigated for their ability to suppress *Colletotrichum* spp. through direct antagonism, resource competition, and stimulation of plant defense responses.^21-24^ Effective implementation requires potent strain selection, field-relevant evaluation, and stable formulations suitable for seed treatment, soil amendment, foliar application, or postharvest protection.^23^ ^,^ ^25-27^ Therefore, beneficial fungi represent an important component of integrated disease management strategies for anthracnose.

Among these fungal biocontrol agents, *Trichoderma* has received particular attention because of its ecological adaptability, rapid growth, rhizosphere competence, and broad functional versatility.^28-30^ Its suppressive activity against *Colletotrichum* is associated with several complementary mechanisms, including mycoparasitism, antibiosis, competition for nutrients and space, and induction of plant systemic resistance; however, these mechanisms are discussed in detail in later sections of this review.^31-33^ By briefly positioning *Trichoderma* within the broader context of biological control, this Introduction provides the rationale for focusing on its potential while avoiding repetition of mechanistic details presented in Section 4.

*Trichoderma* spp. demonstrate antagonistic activity against *Colletotrichum* spp. through multiple complementary mechanisms. Their hyphae can recognize and coil around pathogen structures, while secreted chitinases and *β*-1,3-glucanases degrade fungal cell walls.^34^ ^,^ ^35^ *Trichoderma* spp. also produce antifungal secondary metabolites, including peptaibols, gliotoxin, and viridin, which can inhibit sporulation and mycelial expansion of *C. gloeosporioides* and *C. acutatum*.^6^ ^,^ ^7^ ^,^ ^19^ In vitro studies frequently report substantial growth inhibition, whereas greenhouse and field applications show variable but promising reductions in lesion size, disease incidence, and yield loss across crops such as chili, mango, papaya, strawberry, and coffee.^20^ ^,^ ^36^ Collectively, these findings indicate that *Trichoderma* spp. have multi-mechanistic potential for suppressing *Colletotrichum* spp., although their efficacy remains context-dependent under field conditions.

In addition to their ecological adaptability, *Trichoderma* spp. contribute to soil microbial communities by enhancing nutrient cycling, promoting root development, and improving plant stress tolerance through phytohormone modulation.^10^ ^,^ ^37^ Their ability to colonize root surfaces and persist in the rhizosphere supports sustained interactions with crops and improves competitiveness in soil environments.^29^ ^,^ ^38^ The combination of broad distribution, species diversity, and functional versatility positions *Trichoderma* spp. as important microorganisms in biological control strategies aimed at reducing reliance on synthetic fungicides and improving agroecosystem resilience.^23^ ^,^ ^31^

Although several reviews have addressed either the biology of *Colletotrichum* spp. or the general biocontrol potential of *Trichoderma* spp., the available literature remains relatively fragmented. Many previous studies emphasize taxonomy, infection biology, or disease management separately, while fewer synthesize how recent advances in *Colletotrichum* biology can be directly linked to the multi-mechanistic suppressive actions of *Trichoderma*. In particular, limited reviews have critically integrated mycoparasitism, antibiosis, nutrient competition, induced systemic resistance, and plant growth promotion with application evidence from laboratory, greenhouse, field, and postharvest systems. Therefore, the novelty of this review lies in providing an integrated pathogen–biocontrol framework that connects *Colletotrichum* infection strategies with *Trichoderma*-mediated suppression mechanisms and evaluates their practical relevance for sustainable anthracnose management.

This review aims to provide an integrated and updated synthesis of *Trichoderma*-based management of diseases caused by *Colletotrichum* spp. by linking pathogen biology with biocontrol mechanisms and practical application outcomes. Specifically, this review aims to: (i) synthesize recent advances in the taxonomy, infection biology, and epidemiology of *Colletotrichum* species affecting major agricultural crops; (ii) critically examine the antagonistic and plant-mediated mechanisms employed by *Trichoderma* spp., including mycoparasitism, antibiosis, competition for nutrients and space, and induction of host systemic resistance; and (iii) evaluate the efficacy and limitations of *Trichoderma*-based applications across laboratory, greenhouse, field, and postharvest systems in comparison with conventional chemical fungicides. By integrating these aspects, this review highlights current knowledge gaps, implementation challenges, and future opportunities for developing *Trichoderma*-based strategies as part of sustainable anthracnose management.

## 2. Literature search strategy and review methodology

This review was conducted using a structured narrative approach to synthesize current knowledge on the role of *Trichoderma* spp. in managing plant diseases caused by *Colletotrichum* spp. Relevant literature was collected from major scientific databases, including Scopus, Web of Science, PubMed, ScienceDirect, SpringerLink, MDPI, Frontiers, and Google Scholar. The search terms included combinations of keywords such as “*Trichoderma*”, “*Colletotrichum*”, “anthracnose”, “biological control”, “mycoparasitism”, “antibiosis”, “volatile organic compounds”, “secondary metabolites”, “induced systemic resistance”, “plant growth promotion”, “rhizosphere competition”, and “postharvest disease management”. Literature published between 2000 and 2025 was considered, with priority given to recent studies from 2020–2025 to capture updated advances in taxonomy, molecular mechanisms, omics-based approaches, and applied biocontrol strategies.

Studies were included if they provided relevant information on *Colletotrichum* taxonomy, infection biology, epidemiology, or disease management. Experimental studies were also included when they evaluated *Trichoderma*-mediated suppression under laboratory, greenhouse, field, or postharvest conditions. Priority was given to studies reporting identifiable *Trichoderma* and *Colletotrichum* species, clear antagonistic mechanisms, or measurable disease suppression outcomes.

Review articles and book chapters were used to support broader conceptual frameworks, historical context, and established mechanisms. Studies were excluded when they focused on unrelated pathogens, lacked relevance to biological control, provided insufficient methodological detail, or reported unclear microbial identification. The selected literature was synthesized thematically into four components: *Colletotrichum* biology and pathogenicity, *Trichoderma* biology and ecology, mechanisms of disease suppression, and application evidence across production systems. This approach enabled critical integration of mechanistic and applied evidence while identifying limitations and future research priorities for sustainable anthracnose management.

## 3. Biology and pathogenicity of *Colletotrichum*

The cycle of *Colletotrichum* starts when conidia attach to a host surface, germinate, and produce germ tubes in moist environments.^3^ ^,^ ^39^ These germ tubes then develop into appressoria, which generate strong pressure to break through the plant cuticle.^4^ ^,^ ^5^ Once inside, the fungus enters a biotrophic stage, forming primary hyphae that grow within still-living host cells.^40^ ^,^ ^41^ Later, it shifts to a necrotrophic phase, producing secondary hyphae that kill plant tissue and expand the infection.^37^ ^,^ ^42^ This tightly regulated transition from biotrophy to necrotrophy is a key determinant of pathogenic success and disease severity in *Colletotrichum* infections.

**Figure 1.**
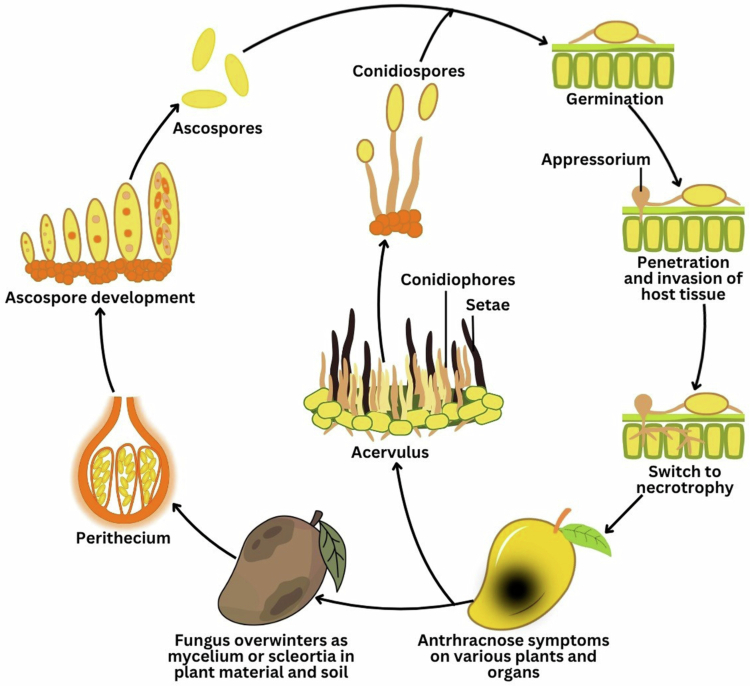
Life cycle and infection process of *Colletotrichum* spp. causing anthracnose disease. The diagram illustrates the major developmental and epidemiological stages of *Colletotrichum*, including ascospore release, conidial production, conidial germination, appressorium formation, host penetration, necrotrophic transition, anthracnose symptom development, and acervuli formation. It also shows pathogen overwintering as mycelium or sclerotia in plant debris and soil, which supports survival between growing seasons. The circular arrows indicate the repeated infection cycle and the capacity of the pathogen to persist under favorable conditions. This figure highlights key intervention points for disease management, including inhibition of spore germination, appressorium formation, host penetration, sporulation, and overwintering survival.

During necrotrophy, the pathogen forms acervuli, structures filled with conidia that serve as new inoculum sources.^30^ ^,^ ^43^ These conidia spread via rain splash, wind, or direct contact, enabling rapid infection of nearby plant tissues.^30^ ^,^ ^44^ In favorable humidity, conidia germinate again and allow the fungus to repeat infection cycles within the same season.^27^ ^,^ ^45^ Some species also generate sclerotia or persistent mycelia, which help them remain viable when no host is available.^44^ ^,^ ^45^ The integration of reproductive efficiency, dispersal capacity, and survival strategies constitutes a pivotal component of the *Colletotrichum* life cycle (Figure 1), collectively underpinning its long-term persistence and epidemic potential in agricultural ecosystems.

The life cycle of *Colletotrichum* reflects a specialized infection strategy that integrates morphological differentiation with environmental responsiveness to support host colonization and disease spread.^32^ ^,^ ^40^ ^,^ ^44^ Key stages, including conidial germination, appressorium formation, host penetration, and the transition from biotrophy to necrotrophy, are tightly regulated and coordinated with host physiological conditions.^32^ ^,^ ^45^ This hemibiotrophic lifestyle allows the pathogen to initially evade host defenses before causing extensive tissue damage during later infection stages.^45^ Reproductive structures such as acervuli and perithecia further enable dissemination and persistence under fluctuating environmental conditions.^46^ Understanding this dynamic life cycle is essential for identifying critical intervention points for disease management and biocontrol strategies.

The life cycle of *Colletotrichum* supports its success as a plant pathogen by integrating efficient infection, reproduction, and environmental adaptation.^5^ ^,^ ^20^ Melanized appressoria enable direct host penetration independent of natural openings, while the transition to necrotrophy enhances nutrient acquisition and symptom development.^4^ ^,^ ^40^ The dual reproductive strategy, involving asexual conidia and sexual ascospores, increases genetic diversity and epidemiological resilience across cropping systems.^44^ ^,^ ^45^ In addition, overwintering as mycelium or sclerotia ensures persistence between growing seasons and contributes to recurrent disease outbreaks.^47^ Collectively, these traits highlight the complexity of the *Colletotrichum* life cycle and support the need for integrated management strategies targeting multiple developmental stages.

*Colletotrichum* spp. infect plants through direct cuticle penetration using melanized appressoria that generate high turgor pressure.^15^ ^,^ ^47^ After entry, the fungus follows a hemibiotrophic lifestyle, initially colonizing living cells before shifting to a necrotrophic phase that causes extensive tissue damage.^15^ ^,^ ^45^ Pathogenicity is mediated by cell wall-degrading enzymes (CWDEs), effectors, and secondary metabolites that suppress host defenses.^43^ These processes produce anthracnose symptoms such as necrotic lesions, chlorosis, and fruit rot, leading to significant yield losses under favorable environmental conditions.^40^ ^,^ ^48^ Collectively, specialized infection structures and molecular virulence mechanisms underpin the strong pathogenic potential of *Colletotrichum* in agricultural systems.

Virulence in *Colletotrichum* spp. is a multifactorial trait involving coordinated physical, biochemical, and molecular mechanisms that enable host penetration, colonization, and disease progression.^15^ ^,^ ^49^ A key determinant is the formation of melanized appressoria, which generate high turgor pressure through osmolyte accumulation and allow direct penetration of the host cuticle.^6^ ^,^ ^40^ ^,^ ^44^ Melanin deposition is essential for maintaining this pressure, and disruption of melanin biosynthesis genes such as *PKS1* can result in loss of pathogenicity.^40^ ^,^ ^41^ ^,^ ^43^ Beyond mechanical force, appressoria also function as localized sites for effector and enzyme secretion during host invasion.^15^ ^,^ ^28^ Collectively, appressoria act as multifunctional infection structures that integrate physical penetration with biochemical support during early infection.

CWDEs represent another major class of virulence factors in *Colletotrichum*, including cutinases, pectinases, cellulases, and hemicellulases that dismantle plant structural barriers during infection.^43^ ^,^ ^50^ Cutinases are particularly important during early infection because they degrade the plant cuticle, and mutants deficient in cutinase activity show reduced penetration and virulence.^51^ ^,^ ^52^ During necrotrophy, pectinases and cellulases promote tissue maceration, nutrient acquisition, and lesion development.^11^ ^,^ ^48^ CWDEs expression is tightly regulated and induced by host-derived signals, reflecting their adaptive role in pathogenesis.^44^ ^,^ ^53^ Therefore, CWDEs function as regulated enzymatic tools that enable both host penetration and progressive tissue colonization.

*Colletotrichum* spp. secrete a diverse set of effector proteins that manipulate host cellular processes and suppress plant immune responses.^15^ ^,^ ^53^ ^,^ ^54^ These effectors are primarily expressed during the biotrophic phase, allowing the pathogen to maintain host cell viability while limiting recognition by pattern recognition receptors (PRRs).^55^ Some effectors suppress reactive oxygen species (ROS) production and interfere with salicylic acid (SA)-mediated signaling, thereby delaying immune activation.^43^ Comparative genomic studies indicate that effector repertoires vary among species, contributing to host specificity and differential virulence.^56^ Collectively, effector-mediated immune modulation is an important strategy that enables *Colletotrichum* to establish infection and overcome host defenses.

Secondary metabolites also function as important chemical virulence factors in *Colletotrichum*, contributing to host cell death and competitive fitness within plant tissues.^4^ ^,^ ^43^ ^,^ ^57^ These metabolites include phytotoxic compounds that disrupt membrane integrity, inhibit photosynthesis, or induce programmed cell death, thereby facilitating the transition from biotrophy to necrotrophy.^4^ ^,^ ^53^ ^,^ ^54^ The hemibiotrophic lifestyle of *Colletotrichum* is closely linked to the temporal regulation of these toxins, ensuring host survival during early infection and aggressive tissue destruction during later stages.^6^ ^,^ ^48^ Such precise regulation reflects a sophisticated virulence strategy optimized for maximal nutrient extraction and disease severity.

Virulence in *Colletotrichum* is regulated by signaling pathways and transcriptional networks, including MAP kinase cascades and cAMP-dependent systems, which coordinate development, stress tolerance, and pathogenicity.^4^ ^,^ ^15^ ^,^ ^44^ These pathways control appressorium formation, enzyme secretion, and effector expression in response to host and environmental cues, and disruption of key genes often reduces virulence.^6^ ^,^ ^15^ Environmental factors also modulate disease development, with temperature influencing spore germination, infection structure formation, and enzymatic activity.^43^ ^,^ ^53^ Optimal pathogenicity typically occurs at 25–30°C, whereas suboptimal conditions can suppress infection and disease progression.^15^ ^,^ ^48^ Together, these regulatory and environmental factors determine the efficiency and severity of *Colletotrichum* infection in agricultural systems.

Moisture and relative humidity are critical drivers of disease outbreaks, as *Colletotrichum* conidia require free water or prolonged leaf wetness periods for germination and successful host penetration.^20^ ^,^ ^58^ High humidity promotes conidial adhesion to plant surfaces and facilitates appressorium differentiation, while extended dew periods allow sufficient time for penetration peg formation and cuticle breach.^53^ ^,^ ^59^ Rainfall events further enhance disease spread by splash dispersal of conidia from infected tissues to healthy plant organs, accelerating epidemic development at the field scale.^6^ ^,^ ^58^ As a result, anthracnose diseases are particularly severe in tropical and subtropical agroecosystems characterized by frequent rainfall and high atmospheric moisture.^28^

Light and photoperiod also influence *Colletotrichum* pathogenicity by regulating sporulation, melanin synthesis, and infection structure differentiation.^41^ ^,^ ^60^ Light-dependent signaling pathways modulate appressorium development and stress tolerance, enabling the fungus to synchronize infection with favorable environmental windows.^3^ ^,^ ^44^ Ultraviolet radiation can suppress conidial viability; however, melanized appressoria provide protection against UV-induced damage, indirectly enhancing survival and infection success under field conditions.^4^ ^,^ ^61^ Thus, light acts not only as a stress factor but also as a regulatory signal shaping pathogen behavior.

Finally, climate variability and climate change are emerging as major factors shaping *Colletotrichum* disease dynamics through altered temperature regimes, rainfall patterns, and extreme weather events.^5^ ^,^ ^62^ Increased humidity and warmer temperatures can extend the geographic range and seasonal activity of *Colletotrichum* species, leading to more frequent and severe anthracnose outbreaks.^4^ ^,^ ^48^ Such environmental shifts may also disrupt existing host resistance mechanisms, necessitating integrated disease management strategies that incorporate biological control agents such as *Trichoderma* to buffer environmentally driven disease risks.^30^ ^,^ ^63^ Collectively, these factors highlight the strong dependency of *Colletotrichum* pathogenicity on environmental context.

The interaction between *Colletotrichum* spp. and their plant hosts is highly dynamic and reflects a continuous molecular arms race between fungal virulence strategies and host immune surveillance systems.^15^ ^,^ ^20^ ^,^ ^64^ Plants initially recognize *Colletotrichum* through conserved pathogen-associated molecular patterns (PAMPs), such as chitin fragments derived from fungal cell walls, which are detected by pattern recognition receptors (PRRs) located on the plant cell surface.^4^ ^,^ ^65^ This recognition triggers PAMP-triggered immunity (PTI), leading to early defense responses including calcium influx, ROS production, and transcriptional activation of defense-related genes.^6^ ^,^ ^64^ However, *Colletotrichum* can suppress or evade PTI during its biotrophic phase, allowing it to establish intracellular primary hyphae while maintaining host cell viability.^15^ ^,^ ^39^ Thus, the ability of *Colletotrichum* to modulate early host immune responses is a critical determinant of successful colonization and disease establishment.

To overcome host immunity, *Colletotrichum* secretes effector proteins that interfere with plant defense signaling and cellular homeostasis, a strategy central to successful colonization.^17^ ^,^ ^51^ These effectors can suppress ROS accumulation, inhibit callose deposition, and modulate hormone signaling pathways such as SA, thereby dampening immune responses during early infection.^4^ ^,^ ^43^ ^,^ ^65^ In resistant hosts, some of these effectors are recognized by intracellular nucleotide-binding leucine-rich repeat (NLR) proteins, activating effector-triggered immunity (ETI), which is often associated with a hypersensitive response (HR) that restricts fungal growth.^39^ ^,^ ^64^ The balance between effector-mediated suppression and host recognition determines whether infection remains localized or progresses to disease.

As *Colletotrichum* transitions from biotrophy to necrotrophy, host–pathogen interactions shift dramatically, with plant defense responses becoming less effective against aggressive tissue degradation.^28^ ^,^ ^54^ During this phase, the pathogen induces host cell death through toxins, CWDEs, and oxidative stress, exploiting the dead tissue as a nutrient source.^4^ ^,^ ^48^ While programmed cell death can be an effective defense against biotrophs, it paradoxically benefits necrotrophic pathogens like *Colletotrichum* during later infection stages.^53^ ^,^ ^66^ This lifestyle plasticity allows *Colletotrichum* to manipulate host defenses temporally, minimizing resistance during early colonization and maximizing nutrient release during necrosis.^15^ Therefore, the strategic shift to necrotrophy represents a critical phase that enhances pathogen fitness and drives severe disease development.

Plant hormonal signaling networks play a central role in regulating defense outcomes during *Colletotrichum* infection.^4^ ^,^ ^53^ ^,^ ^66^ SA–mediated defenses are primarily associated with resistance to biotrophic pathogens and are most active during early infection, whereas jasmonic acid (JA) and ethylene (ET) signaling pathways dominate responses against necrotrophic pathogens.^45^ ^,^ ^66^ ^,^ ^67^ *Colletotrichum* exploits hormonal crosstalk by suppressing SA signaling during biotrophy and inducing JA/ET responses during necrotrophy, thereby fine-tuning host immunity to favor disease development.^11^ ^,^ ^43^ ^,^ ^67^ This hormonal manipulation underscores the complexity of host–pathogen interactions beyond simple defense activation.

In addition to local responses, plants can activate systemic defense mechanisms, including systemic acquired resistance (SAR) and induced systemic resistance (ISR), which enhance resistance in distal tissues.^41^ ^,^ ^67^ While SAR is typically SA-dependent and associated with pathogen infection, ISR is commonly activated by beneficial microbes such as *Trichoderma*, priming the plant for faster and stronger defense responses upon *Colletotrichum* challenge.^20^ ^,^ ^68^ These systemic responses involve epigenetic modifications, transcriptional reprogramming, and metabolic adjustments that collectively increase host resilience.^4^ ^,^ ^67^ Understanding these multilayered defense strategies provides a critical framework for integrating biological control approaches into sustainable disease management.

## 4. Overview of *Trichoderma*

*Trichoderma* is a widely distributed genus of filamentous ascomycete fungi within the family Hypocreaceae, commonly found in soil and plant-associated environments.^37^ ^,^ ^69^ Its species are characterized by rapid growth, high sporulation capacity, and strong metabolic versatility, enabling effective competition in rhizosphere ecosystems.^30^ ^,^ ^70^ ^,^ ^71^ Morphologically, *Trichoderma* exhibits branched conidiophores with whorled phialides that produce green, easily dispersed conidia.^72^ ^,^ ^73^ Advances in molecular phylogenetics have revealed significant cryptic diversity, with species identification now relying on multi-locus sequencing rather than morphology alone. Thus, the combination of morphological adaptability and genetic diversity underpins the ecological success and functional versatility of *Trichoderma* in diverse environments.

The morphological and structural characteristics of *Trichoderma* play a fundamental role in its effectiveness as a biological control agent against plant pathogens such as *Colletotrichum*. Both macroscopic colony development and microscopic features including hyphal architecture, conidiophore organization, and spore formation are closely linked to its ecological adaptability and antagonistic capacity.^52^ Rapid growth, high sporulation rates, and extensive hyphal branching enable *Trichoderma* to efficiently colonize substrates and outcompete pathogenic fungi in diverse environments.^74^ In addition, these structural traits facilitate direct interactions with pathogens, including attachment, coiling, and penetration during mycoparasitism.^24^ Therefore, understanding the morphological and microscopic features of *Trichoderma* provides essential insight into its functional role in disease suppression.

**Figure 2.**
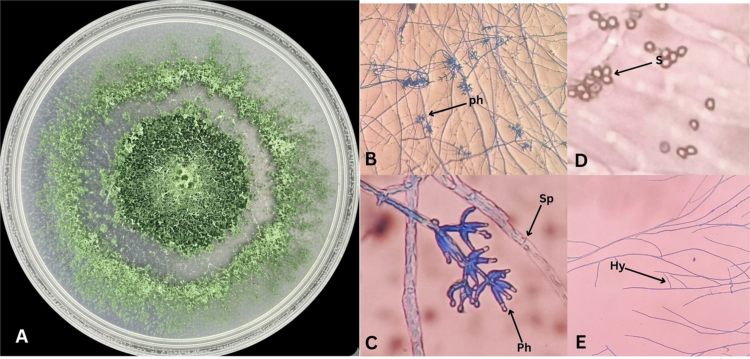
Macroscopic and microscopic characteristics of *Trichoderma* spp. (A) Macroscopic colony morphology showing dense green sporulation and rapid radial growth on culture medium. (B–C) Microscopic structures showing branched conidiophores and phialides, which are associated with conidial production. (D) Conidia/spores showing rounded to oval propagules involved in fungal dispersal. (E) Hyphal structures showing filamentous growth typical of *Trichoderma*. Abbreviations: ph/Ph, phialide; S, spores or conidia; Sp, septae; Hy, hyphae. These morphological characteristics are associated with rapid colonization, high reproductive capacity, and competitive ability, which support the ecological fitness and biocontrol potential of *Trichoderma* spp. (Photographs courtesy of Rendra Adhitya).

As shown in Figure 2, *Trichoderma* spp. exhibit distinct morphological features at both colony and cellular levels that support their biocontrol efficiency. Dense and rapidly expanding colony growth reflects strong competitive ability, allowing *Trichoderma* spp. to occupy ecological niches and limit pathogen establishment.^75^ ^,^ ^76^ Microscopically, highly branched hyphae, specialized conidiophores, and abundant spores enhance their capacity for reproduction and dispersal.^27^ ^,^ ^30^ These traits are associated with key antagonistic mechanisms, including rhizosphere colonization, physical interaction with pathogen hyphae, and production of dispersal propagules.^24^ Structural plasticity across developmental stages further highlights their adaptability under varying environmental conditions.^69^ ^,^ ^76^ Collectively, these morphological and microscopic traits support the role of *Trichoderma* spp. as effective and versatile biocontrol agents in agricultural systems.

Ecologically, *Trichoderma* functions primarily as a saprotroph and opportunistic symbiont, colonizing organic matter and plant roots without causing disease.^71^ ^,^ ^72^ The rhizosphere serves as a key habitat where it competes with phytopathogens for nutrients and space while utilizing root exudates to support growth and activity.^26^ In addition, *Trichoderma* interacts with host plants by modulating physiological processes and enhancing plant performance.^63^ ^,^ ^68^ Its ability to tolerate fluctuations in pH, temperature, and soil moisture further supports its persistence across diverse environments.^72^ ^,^ ^77^ Thus, this ecological adaptability enables *Trichoderma* to function effectively as a resilient and competitive biocontrol agent in agroecosystems.

At the biological level, *Trichoderma* spp. possess a formidable array of enzymatic and secondary metabolic functions that support their antagonistic behavior towards plant pathogens like *Colletotrichum*.^78^ ^,^ ^79^ These fungi release significant amounts of hydrolytic enzymes, such as chitinases, glucanases, and proteases, which allow them to break down fungal cell walls and exploit other fungi as nutrient sources through mycoparasitism.^63^ In addition to their enzymatic functions, *Trichoderma* generates a diverse range of bioactive secondary metabolites, including peptaibols, polyketides, and volatile organic compounds (VOCs), that inhibit pathogen growth and disrupt pathogen signaling.^78^ ^,^ ^80^ The synchronized regulation of these characteristics enables *Trichoderma* to swiftly react to the presence of competing microorganisms in its surroundings.

In addition to its direct antagonistic properties, *Trichoderma* significantly contributes to plant growth enhancement and immune modulation, positioning it as an ideal candidate for integrated disease management approaches.^63^ ^,^ ^79^ The colonization by *Trichoderma* can improve nutrient uptake efficiency, enhance root structure, and promote plant growth through the synthesis of auxin-like substances and the modulation of nutrient solubilization processes.^76^ Notably, *Trichoderma* triggers systemic resistance in plants by activating JA and ET signaling pathways, thus preparing host defenses against pathogens such as *Colletotrichum*.^56^ ^,^ ^68^ This dual function as both a microbial antagonist and a plant defense enhancer sets *Trichoderma* apart from numerous other biocontrol agents.

From an applied ecological standpoint, *Trichoderma* has been significantly developed into commercial biocontrol products owing to its safety, compatibility with the environment, and broad-spectrum efficacy.^63^ Its capacity to form stable populations in both the rhizosphere and phyllosphere enables ongoing suppression of pathogens in field settings, even amidst complex microbial communities.^37^ ^,^ ^81^ Additionally, *Trichoderma* engages in synergistic interactions with beneficial soil microbiota, enhancing soil health and resilience while decreasing dependence on chemical fungicides.^35^ In summary, the biological, ecological, and functional characteristics of *Trichoderma* highlight its pivotal role in the sustainable management of plant diseases caused by *Colletotrichum* spp.

## 5. Mechanisms of *Trichoderma* in disease suppression

### 5.1. Mycoparasitism

Mycoparasitism in *Trichoderma* spp. begins with early recognition of fungal targets through molecular signaling before physical contact.^82^ This recognition is mediated partly by lectin–carbohydrate interactions, particularly chitin-derived molecules that guide directional hyphal growth toward *Colletotrichum* spp.^83^ Upon contact, *Trichoderma* hyphae attach, coil, and form appressorium-like structures that facilitate adhesion and penetration.^84^ Transcriptomic analyzes further reveal stage-specific activation of genes related to signaling, transport, and cell wall degradation.^9^ ^,^ ^82^ Together, early recognition and targeted attachment represent critical steps that determine the efficiency of mycoparasitic interaction and subsequent pathogen suppression.

Enzymatic degradation is a central component of *Trichoderma* mycoparasitism, targeting structural polymers such as chitin, glucans, and proteins in pathogen cell walls.^85^ *Trichoderma* spp. produces chitinases (GH18), glucanases, and proteases that act synergistically to degrade fungal cell walls, leading to hyphal collapse and cytoplasmic leakage.^71,85–87^ Chitinase activity is induced by pathogen-derived signals, enhancing degradation efficiency during interaction.^50^ ^,^ ^86^ While proteases disrupt structural proteins and virulence-associated factors, facilitating deeper penetration.^87^ Together, these enzymatic activities constitute a highly coordinated system that enables effective degradation of pathogen structures and reinforces *Trichoderma* antagonistic capacity.

Mycoparasitic interactions between *Trichoderma* and phytopathogenic fungi such as *Colletotrichum* involve a highly coordinated sequence of recognition, attachment, and enzymatic degradation processes.^10^ These interactions are mediated by chemical signaling and surface molecule recognition that guide *Trichoderma* hyphae toward their fungal targets.^46^ Upon close proximity, *Trichoderma* establishes physical contact and initiates structural modifications that facilitate host invasion.^87^ This process is further supported by the secretion of lytic enzymes and secondary metabolites that weaken pathogen cell walls.^52^ Understanding these multi-layered interactions is essential for elucidating the mechanistic basis of *Trichoderma* mediated biocontrol.^31^

*Trichoderma* hyphae actively recognize and coil around the hyphae of target fungi, forming tight physical associations characteristic of mycoparasitic behavior.^9^ ^,^ ^50^ ^,^ ^88^ This coiling facilitates localized secretion of hydrolytic enzymes, such as chitinases and glucanases, which degrade the pathogen cell wall and contribute to structural collapse.^50^ Close contact also enables efficient delivery of antifungal metabolites, enhancing inhibitory effects on pathogen growth and development.^52^ Together, direct physical interaction and biochemical attack are central to the biocontrol activity of *Trichoderma* against fungal pathogens.^32^ ^,^ ^71^

This type of overgrowth is generally preceded at the microscopic scale by hyphal coiling, development of appressorium-like structures, and localized invasion of the host cell wall.^9^ ^,^ ^10^ ^,^ ^38^ Because *Colletotrichum* cell walls are rich in chitin and *β*-glucans, they are primary targets of *Trichoderma*-produced chitinases and glucanases. ^5^ ^,^ ^89^

### 5.2. Antibiosis

Antibiosis in *Trichoderma* involves the production of diverse antifungal metabolites that inhibit *Colletotrichum* spp. growth and disrupt cellular integrity.^32^ ^,^ ^37^ Compounds such as gliotoxin induce oxidative stress and inhibit enzyme function, while viridine and viridiol interfere with respiration and membrane-associated processes.^48^ ^,^ ^90^ Peptaibols form ion channels in fungal membranes, causing depolarization and cell lysis, thereby limiting key infection processes such as appressorium formation.^91^ ^,^ ^92^ The production of these metabolites is tightly regulated by environmental signals and global regulatory networks.^23^ Collectively, these chemically mediated interactions highlight antibiosis as a critical mechanism underlying *Trichoderma* driven suppression of fungal pathogens.

Recent studies highlight the role of VOCs such as 2-pentyl furan and dimethyl disulfide, which inhibit *Colletotrichum* through membrane disruption and interference with respiration.^52^ Non-volatile metabolites, including polyketides and pyrones such as 6-pentyl-*α*-pyrone, accumulate locally and enhance antifungal activity.^71^ ^,^ ^93^ In vitro assays consistently demonstrate strong inhibition, often exceeding 70% reduction in pathogen growth under dual culture conditions.^94^ These findings confirm antibiosis as a major chemical mechanism in *Trichoderma*-mediated suppression.

### 5.3. Competition for nutrients and space

Efficient rhizosphere colonization enables *Trichoderma* to outcompete pathogens by occupying ecological niches and rapidly utilizing root-derived nutrients.^47^ ^,^ ^85^ Extensive hyphal branching allows rapid exploration of soil microenvironments, forming physical exclusion zones that restrict pathogen establishment near roots.^47^ ^,^ ^95^ This competitive advantage reduces access of *Colletotrichum* to infection sites and essential resources. As a result, pathogen colonization is suppressed before infection can occur.

*Trichoderma* expands aggressively across the substrate, occupying more space than *Colletotrichum* and restricting pathogen access to nutrients and colonization sites.^32^ ^,^ ^38^ ^,^ ^69^ Its dense hyphal network and rapid radial growth create a competitive barrier that limits pathogen expansion and reduces infection potential.^38^ ^,^ ^73^ In addition, depletion of available carbon sources and micronutrients, such as iron, further suppresses pathogen growth by constraining metabolic activity.^70^ ^,^ ^71^ This spatial dominance can also reduce contact between the pathogen and host tissues, indirectly limiting disease establishment.^14^ ^,^ ^87^ Collectively, these competitive interactions highlight resource acquisition and niche occupation as key determinants of *Trichoderma*-mediated disease suppression.

Iron competition plays a critical role, as *Trichoderma* produces siderophores that chelate Fe³⁺, limiting its availability to competing pathogens.^33^ ^,^ ^96^ Under iron-limited conditions, hydroxamate-type siderophores enhance nutrient acquisition efficiency and suppress pathogen metabolism.^47^ In parallel, *Trichoderma* exhibits strong carbon scavenging capacity, utilizing diverse substrates and rapidly depleting energy sources required for pathogen growth.^33^ The production of organic acids further modifies the microenvironment, enhancing nutrient solubilization while disadvantaging competing fungi.

### 5.4. Induced systemic resistance (ISR) and plant growth promotion

*Trichoderma* induces systemic resistance by activating plant defense genes, including pathogenesis-related (PR) proteins and enzymes of the phenylpropanoid pathway.^81^ Upregulation of phenylalanine ammonia-lyase (PAL) promotes synthesis of lignin, flavonoids, and phytoalexins that strengthen structural and chemical defenses.^97^ ^,^ ^98^ PR proteins such as *β*-1,3-glucanases and thaumatin-like proteins directly inhibit fungal pathogens and amplify defense signaling.^97^ These responses collectively enhance resistance against *Colletotrichum* infection.

ISR is primarily mediated through JA and ET signaling pathways, which regulate defense gene expression and antimicrobial compound production.^68^ ^,^ ^99^ SA signaling is also primed, enabling rapid activation of systemic acquired resistance upon pathogen challenge.^67^ In addition, *Trichoderma* improves plant growth by enhancing nutrient uptake, photosynthetic efficiency, and stress tolerance.^38^ ^,^ ^98^ These combined effects strengthen plant resilience and reduce disease severity under pathogen pressure.

## 6. *Trichoderma*-based strategies for the management of *Colletotrichum* diseases

Application studies of *Trichoderma* spp. against *Colletotrichum* spp. indicate promising antagonistic potential across laboratory, greenhouse, field, and postharvest systems, although the strength of evidence differs among experimental scales.^32^ ^,^ ^100^ In vitro assays, particularly dual-culture and metabolite-based inhibition tests, frequently report high suppression rates linked to mycoparasitism and antibiosis.^81^ However, greenhouse and field studies introduce greater ecological complexity, including variation in host genotype, pathogen pressure, native microbial competition, and abiotic conditions.^10^ These factors can reduce or modify biocontrol performance under practical conditions. Postharvest and in planta studies further suggest that *Trichoderma* metabolites may delay lesion development and anthracnose progression, although outcomes remain dependent on strain identity and application strategy.^95^ Therefore, current evidence supports *Trichoderma* spp. as promising but context-dependent biocontrol agents rather than direct replacements for fungicides across all systems.

The compiled evidence indicates that *Trichoderma*-based applications can suppress *Colletotrichum* spp. across laboratory, greenhouse, field, and postharvest systems, although performance remains context-dependent.^79^ ^,^ ^101^ Laboratory studies provide strong evidence of direct antagonism through mechanisms such as mycoparasitism and antibiosis, whereas greenhouse and field conditions introduce environmental variability that can influence efficacy.^37^ ^,^ ^102^ Differences in strain specificity, host plant interactions, pathogen pressure, and application methods further contribute to variation in disease control outcomes.^83^ Overall, available evidence suggests that *Trichoderma* spp. can reduce disease incidence, lesion development, and pathogen growth when appropriately applied.^58^ These findings support the integration of *Trichoderma* into sustainable disease management programs, although optimization of strain selection, formulation, and field application strategies remains essential to achieve consistent and scalable results.

Beyond efficacy, the successful implementation of *Trichoderma*-based biocontrol depends on overcoming practical and technological constraints related to formulation stability, shelf life, and field adaptability.^37^ Environmental conditions, including temperature, humidity, and soil composition, can influence microbial survival, colonization efficiency, and metabolite production, thereby affecting overall performance.^10^ ^,^ ^63^ Large-scale application also requires standardized formulations that maintain viability and activity during storage and distribution, which remains a key challenge for commercialization.^23^

Advances in formulation technologies, including encapsulation, carrier-based systems, and integration with organic amendments, may enhance stability and field performance.^33^ Combining *Trichoderma* with complementary biocontrol agents or reduced fungicide inputs may also improve consistency through synergistic effects under diverse agricultural conditions.^22^ Taken together, addressing these operational challenges is essential for translating laboratory efficacy into reliable field performance and supporting wider adoption of *Trichoderma*-based solutions in sustainable agriculture.

Table 1 summarizes representative studies evaluating *Trichoderma* spp. against *Colletotrichum* spp. across laboratory, greenhouse, in planta, and field conditions, highlighting variations in application methods, species-specific interactions, and overall disease suppression outcomes. These compiled data illustrate the broad-spectrum biocontrol potential of *Trichoderma* while also emphasizing that efficacy is strongly influenced by environmental complexity, formulation strategy, and host pathogen context. Collectively, the table provides comparative evidence supporting *Trichoderma* as a versatile and sustainable alternative for anthracnose management across multiple agricultural systems.

**Table 1.** *Trichoderma* applications against *Colletotrichum* spp. under in vitro and greenhouse conditions.

Experimental scales	Crop hosts	Methods	*Trichoderma* species	*Collelotrichum* species	Percentage of inhibition	Key results	References
In vitro	Chili	Dual culture + secondary metabolite inhibition	*Trichoderma* spp.	*Colletotrichum* spp.	~45 %	Secondary metabolites inhibited pathogen growth; chili-derived metabolites had highest inhibition	[3]
In vitro and in planta (greenhouse)	Pomelo fruits	Isolate selection + antagonism assay + fruit challenge	*T. asperellum* and *T. harzianum*	*C. gloeosporioides*	~66-68 %	Antagonism in vitro; reduced lesion growth on pomelo fruits	[83]
In planta (greenhouse)	Chili	Plant treatment + disease assessment	*T. harzianum*	*Colletotrichum* spp.	~55 %	Anthracnose inhibition on chili plants under glasshouse conditions	[94]
In vitro	Pathogen culture system	Dual culture + VOC analysis	*T. koningiopsis* PSU3-2	*C. gloeosporioides*	~79.6 %	Inhibition; VOCs implicated in antibiosis	[100]

Although the compiled studies support the suppressive potential of *Trichoderma* against *Colletotrichum* spp., the evidence base remains uneven across experimental systems. Laboratory assays provide clear mechanistic evidence for antagonism, but they do not fully represent the ecological complexity of greenhouse or field environments. Greenhouse and in planta studies provide greater biological relevance, yet they are often limited by specific host–pathogen combinations and controlled conditions.

Field and postharvest studies are more directly applicable to agricultural practice, but they remain comparatively fewer and show variable efficacy depending on strain, formulation, crop system, and environmental context. Consequently, the available evidence supports further development of *Trichoderma* as a complementary component of integrated anthracnose management. However, broader standardized field validation is still required before generalized claims of consistent field efficacy can be made.

### 6.1. Commercial developments and practical adoption of *Trichoderma*-based biofungicides

The commercial adoption of *Trichoderma*-based biofungicides demonstrates that this genus has moved beyond experimental biocontrol research into practical crop protection systems. Several products are marketed globally as biofungicides, bioinoculants, or plant health enhancers, commonly formulated as wettable powders, granules, liquid suspensions, or carrier-based inoculants.^26^ ^,^ ^33^ ^,^ ^103^ Commercial products such as Trianum, RootShield, RootShield PLUS, and other *Trichoderma*-based formulations commonly use strains such as *T. harzianum* T-22, *T. virens* G-41, or related species to suppress soil-borne pathogens, improve root development, and enhance plant vigor.^33^ ^,^ ^103-106^

These products are applied through soil incorporation, seed or transplant treatment, substrate mixing, irrigation systems, foliar application, or postharvest-compatible approaches depending on the formulation and crop system.^26^ ^,^ ^102^ Their practical adoption has been reported in horticultural crops, greenhouse production, ornamentals, nursery systems, vegetables, and some field crops, indicating the broad applicability of *Trichoderma*-based technologies in sustainable agriculture.^24^ ^,^ ^27^ However, commercial performance remains dependent on product quality, viable propagule concentration, formulation stability, storage conditions, application timing, crop species, and local environmental factors.^104^ ^,^ ^107^ Therefore, commercially available *Trichoderma* products should be viewed as promising components of integrated disease management rather than universal standalone replacements for chemical fungicides.

To provide a clearer applied perspective, Table 2 summarizes representative commercially available *Trichoderma*-based biofungicides, including their active microorganisms, formulation types, common application strategies, practical uses, and supporting references. This table illustrates that commercial products are not uniform; they differ in strain composition, delivery system, target production setting, and expected disease-management function. Therefore, the table should be interpreted not only as evidence of commercial availability, but also as an indication that successful adoption depends on matching the product formulation and application method with the crop system, pathogen pressure, and local environmental conditions.

**Table 2.** Representative commercial *Trichoderma*-based biofungicides, active strains, formulations, and practical uses.

Commercial products	Active microorganisms	Common formulation/application	Reported practical use	References
Trianum-*P*/Trianum-G	*T. harzianum* strain T-22	Water-dispersible powder or granular formulation; applied through irrigation, substrate mixing, or soil application	Used for suppression of soil-borne/root pathogens and improvement of root development in horticultural and field crops	[108]
RootShield WP/RootShield G	*T. harzianum* strain T-22	Wettable powder or granular formulation for soil, growing media, transplant, and nursery applications	Used in greenhouse, nursery, ornamental, vegetable, and other crop production systems to suppress root diseases	[96]
RootShield PLUS WP/RootShield PLUS G	*T. harzianum* strain T-22 + *T. virens* strain G-41	Wettable powder or granular formulation; applied to soil, growing media, transplants, and irrigation systems	Designed for broader suppression of root diseases across food crops, ornamentals, turf, landscape plants, and nursery systems	[102,108]

## 7. Challenges and future perspectives

**Figure 3.**
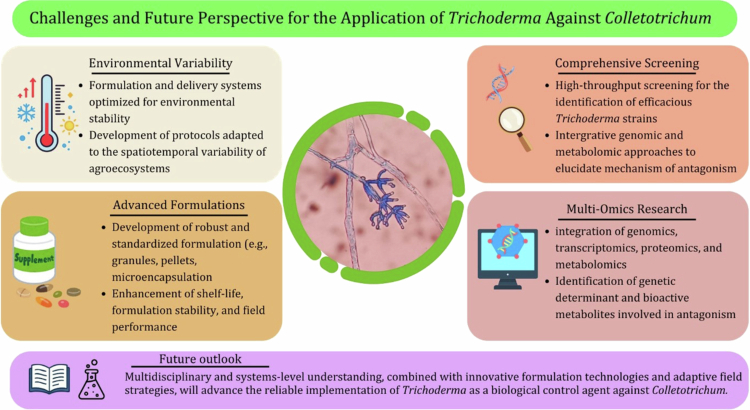
Key challenges and future research directions for *Trichoderma*-based management of *Colletotrichum*-associated diseases. The figure summarizes major constraints limiting the consistent field performance of *Trichoderma* spp., including environmental variability, formulation and delivery limitations, and the need for comprehensive strain screening. It also highlights emerging research directions, such as advanced formulation technologies, high-throughput screening, multi-omics integration, and identification of genetic determinants and bioactive metabolites involved in antagonism. The central image represents morphological features of *Trichoderma* that support strain selection and mechanism-oriented research. Reliable implementation will require the integration of systems-level biological understanding, standardized formulations, and adaptive field strategies tailored to local agroecosystems.

Despite the potential of *Trichoderma* as a biocontrol agent against *Colletotrichum* spp., several challenges limit its consistent performance under field conditions. Environmental variability, including temperature, humidity, soil composition, and microbial community structure, can influence the survival, colonization, and antagonistic activity of *Trichoderma*.^109^ Although laboratory and greenhouse studies often show high efficacy, these results are not always reproducible at field scale because of complex ecological interactions. Therefore, improving the environmental robustness and adaptability of *Trichoderma* strains remains an important research priority.^104^ These challenges and research priorities are summarized in Figure 3, which highlights key constraints and strategic directions for improving the field performance and scalability of *Trichoderma*-based biocontrol systems.

A major limitation of *Trichoderma*-based biocontrol is the inconsistent translation of laboratory and greenhouse efficacy into reliable field performance across agroecological regions.^24^ In vitro assays are conducted under simplified and highly controlled conditions, whereas field environments expose *Trichoderma* spp. to fluctuating temperature, humidity, soil pH, nutrient availability, ultraviolet radiation, and native microbial competition.^74^ These factors can alter spore survival, rhizosphere colonization, antifungal metabolite production, and activation of plant defense responses, thereby reducing disease suppression under certain conditions.^10^ ^,^ ^52^

Moreover, the performance of a given *Trichoderma* strain may vary among crop species, pathogen populations, soil types, and farming systems.^75^ This variability indicates that biocontrol efficacy is not universally transferable across regions. Therefore, region-specific strain selection, standardized multi-location field trials, and locally adapted formulations are needed before *Trichoderma*-based products can be broadly recommended as reliable components of anthracnose management.

Another major challenge is strain specificity and selection. Not all *Trichoderma* isolates exhibit equal biocontrol potential, and their effectiveness can vary depending on the host plant, pathogen species, and environmental context.^85^ This variability highlights the need for systematic and large-scale screening to identify elite strains with broad-spectrum activity and stable performance.^20^ Advances in genomic, transcriptomic, and metabolomic tools offer new opportunities to clarify the molecular basis of antagonism. These approaches may support more precise selection and development of high-performing strains tailored to specific agricultural systems.^109^

Future perspectives for *Trichoderma*-based disease management should integrate multi-omics, microbiome-informed strategies, artificial intelligence-assisted strain selection, and precision agriculture approaches.^110^ Multi-omics tools can identify molecular determinants of mycoparasitism, antibiosis, rhizosphere competence, and plant immune priming, while microbiome-informed strategies may improve the compatibility of *Trichoderma* with beneficial microbial networks.^111^

Artificial intelligence (AI) and machine learning can support predictive strain selection by integrating strain identity, metabolite profiles, host species, pathogen pressure, formulation type, and environmental variables.^81^ ^,^ ^112^ Precision agriculture approaches, including environmental monitoring, disease-risk mapping, and site-specific application, may further optimize the timing and placement of *Trichoderma*-based treatments.^113^ Together, these tools can shift *Trichoderma* biocontrol from empirical screening toward more targeted, data-driven, and field-adapted disease management.

Formulation and application strategies also represent key limitations in the field deployment of *Trichoderma*.^114^ Maintaining long-term viability, shelf stability, and effective delivery remains challenging, particularly under fluctuating environmental conditions.^81^ Innovative formulation technologies, including encapsulation, granules, and carrier-based systems, are needed to improve spore survival, ease of application, and colonization efficiency.^20^ ^,^ ^85^ In addition, optimizing application methods such as seed treatment, soil amendment, foliar spray, and postharvest coating is essential for maximizing biocontrol efficacy across different cropping systems.^109^

Future research should focus on integrating *Trichoderma* into holistic and sustainable disease management frameworks.^46^ This includes combining biocontrol agents with resistant cultivars, cultural practices, and reduced-risk fungicides within integrated pest management (IPM) programs.^88^ Furthermore, multi-omics approaches should be used to better understand *Trichoderma*–plant–pathogen interactions at the systems level, enabling the identification of key regulatory pathways and biomarkers associated with successful biocontrol.^19^ Bridging the gap between laboratory discoveries and field applications through interdisciplinary research and large-scale validation will be essential for developing *Trichoderma* as a reliable and scalable component of anthracnose management.^27^

### 7.1. Emerging trends and research directions

Future research on *Trichoderma*-based management of *Colletotrichum*-associated diseases should move beyond conventional strain screening toward integrated and technology-driven biocontrol development.^24^ Multi-omics approaches, including genomics, transcriptomics, proteomics, metabolomics, and microbiome profiling, offer powerful tools to identify molecular determinants of mycoparasitism, antibiosis, rhizosphere competence, and plant immune priming.^46^ ^,^ ^81^ These approaches can help clarify why some *Trichoderma* strains perform well under laboratory conditions but show variable efficacy in field environments, where host genotype, pathogen pressure, native microbiota, and abiotic stress interact simultaneously.^108^ ^,^ ^115^

In parallel, machine learning and predictive modeling may support the selection of high-performing strains. These approaches can integrate strain identity, metabolite profiles, host species, pathogen species, formulation type, and environmental variables into biocontrol prediction frameworks.^116^ Such predictive tools may reduce trial-and-error screening and improve the reliability of strain selection across different agroecosystems.

Synthetic biology and CRISPR-based genome editing may provide useful tools for improving *Trichoderma* performance by targeting genes related to antifungal metabolite biosynthesis, enzyme secretion, stress tolerance, and plant interaction.^117^ ^,^ ^118^ Recent advances in CRISPR/Cas systems for filamentous fungi and *T. atroviride* demonstrate the technical feasibility of precise genetic manipulation.^118^ ^,^ ^119^ However, ecological safety, regulatory acceptance, and strain stability must be carefully evaluated before agricultural deployment. In addition, genome-guided metabolomics and natural product discovery can accelerate the identification of novel antifungal compounds, including peptaibols, terpenoids, polyketides, and VOCs with potential activity against *Colletotrichum*.^46^ ^,^ ^120^ These metabolite-focused strategies may support the development of improved bioformulations or natural-product-based fungicide alternatives.

Another emerging direction is the development of microbiome-informed and formulation-based strategies that improve the persistence and functional stability of *Trichoderma* under field conditions.^74^ ^,^ ^115^ Because *Trichoderma* interacts with plant-associated microbiomes, future studies should evaluate not only pathogen suppression but also its effects on beneficial microbial networks, soil health, and host physiological performance.^81^ Advanced formulations, including encapsulated spores, carrier-based consortia, bio-organic amendments, and postharvest-compatible coatings, may enhance shelf life, delivery efficiency, and colonization success under variable environmental conditions.^96^ ^,^ ^115^

Large-scale, multi-location field trials are needed to validate laboratory and greenhouse findings under realistic production systems. Supportive regulatory frameworks are also required to ensure safe commercialization and adoption by farmers. Therefore, future progress will depend on integrating molecular innovation, ecological validation, formulation science, and policy support to develop *Trichoderma*-based biocontrol as a more reliable and scalable component of anthracnose management.

## 8. Conclusion

*Trichoderma* spp. represent promising multifunctional biocontrol agents for managing anthracnose diseases caused by *Colletotrichum* spp. Their suppressive potential is supported by multiple mechanisms, including mycoparasitism, antibiosis, nutrient competition, and induction of plant systemic resistance. In addition, their ability to promote plant growth and enhance stress tolerance strengthens their relevance within sustainable disease management systems. However, the available evidence indicates that efficacy is often stronger and more consistent under laboratory and greenhouse conditions than under field or postharvest settings. Therefore, practical application should be viewed as context-dependent and influenced by strain selection, formulation, host plant, pathogen species, and environmental compatibility.

Future progress will rely on integrating advanced approaches such as genomics, metabolomics, transcriptomic, and formulation technologies to optimize strain performance and application strategies. By aligning fundamental research with practical implementation, *Trichoderma* has strong potential to reduce dependence on synthetic fungicides and contribute to resilient, environmentally sustainable disease management systems.

## Data Availability

All relevant data are included in the manuscript.
